# Investigating Flood Risks of Rainfall and Storm Tides Affected by the Parameter Estimation Coupling Bivariate Statistics and Hydrodynamic Models in the Coastal City

**DOI:** 10.3390/ijerph191912592

**Published:** 2022-10-02

**Authors:** Hongshi Xu, Kui Xu, Tianye Wang, Wanjie Xue

**Affiliations:** 1 Yellow River Laboratory, Zhengzhou University, Zhengzhou 450001, China; 2 State Key Laboratory of Hydraulic Engineering Simulation and Safety, Tianjin University, Tianjin 300354, China

**Keywords:** flood risk, parameter estimation, bivariate return period, hydrodynamic model

## Abstract

The public health risk caused by urban floods is a global concern. Flood risks are amplified by the interaction of rainfall and storm tides in coastal cities. In this study, we investigate the flood risks of rainfall and storm tides coupling statistical and hydrodynamic models and evaluate the influence of different parameter estimation methods and bivariate return periods (RPs) on flood risks in the coastal city. The statistical model is used to obtain the bivariate design of rainfall and storm tides with the integration of copula function, most-likely weight function and Monte Carlo simulation method. The bivariate designs are adopted as the input boundaries for the hydrodynamic model established by Personal Computer Storm Water Management Model (PCSWMM), and the flood risk is evaluated by the hydrodynamic model. Subsequently, the influence of different parameter estimation approaches (that is, parametric and non-parametric) and bivariate RPs (that is, co-occurrence RP, joint RP, and Kendall RP) on bivariate designs and flood risks are investigated. With Haikou coastal city in China as the case study, the results show that: (1) Gumbel copula is the best function to describe the correlation structure between rainfall and storm tides for the parametric and non-parametric approaches, and the non-parametric approach is a better fit for the observed data; (2) when the Kendall RP is large (more than 100 years), the flood risk is underestimated with an average of 17% by the non-parametric estimation, and the parametric estimation approach is recommended as it is considered the most unfavorable scenario; (3) the types of bivariate RP have the important impact on the flood risk. When there is no specific application need, the Kendall RP can be adopted as the bivariate design standard of flooding facilities since it can describe the dangerous areas more accurately for multivariate scenario. The results can provide references for reasonable flood risk assessment and flooding facility design in coastal cities.

## 1. Introduction

With the climate change and rapid urbanization, urban floods become more frequent [1,2]. In particular, coastal cities are more susceptible to floods due to the compound effect of rainfall and storm tides [3]. In coastal cities, rainfall is collected by drainage systems, and then flows into the sea and tidal rivers. When the sea level is high, it would have an adverse influence on the drainage capability and could directly cause coastal flooding [4,5]. According to the report by the Intergovernmental Panel on Climate Change (IPCC), extreme rainfall and sea level have rising trends in recent years [6,7,8]. Therefore, there is a great need to investigate the compound flood risks caused by rainfall and storm tides in coastal cities.

In the past 30 years, many scholars have conducted studies on the dependence between rainfall and storm tides at different spatial scales. Ward et al. [9] investigated the dependence between coastal and river flooding by 187 combinations of stations on a global scale; they found that more than 50% of stations show the significant dependence when allowing a time-lag up to 5 days. Zheng et al. [10] applied a bivariate logistic threshold-excess model to quantify the dependence between rainfall and storm surge, where they observed the statistically significant dependence for the majority of stations in the Australian coastline. Wahl et al. [11] demonstrated the increasing risk of compound flooding from storm surge and rainfall for major US cities by Kendall’s τ and copula theory. Lian et al. [12] and Zellou et al. [13] evaluated the joint impact of rainfall and storm tides in Fuzhou of China and Bouregreg estuary of Morocco, respectively, on a local scale. These studies observed the statistically significant dependence of rainfall and storm tides in different coastal cities, and the flood risk would be severely underestimated if the dependence was ignored. In this study, an integrated model coupling the statistical model and hydrodynamic model was used for evaluating flood risk in the coastal city. The statistical model is used to describe the dependence structure of rainfall and storm tides. Commonly used statistical models include copula model [14,15,16] and bivariate logical model [10,17], etc. The copula model provides great flexibility in modeling the dependence structure among random variables, and the joint distribution can be flexibly constructed using a wide variety of copula functions and marginal distributions. Therefore, copula models were applied to derive the bivariate design of rainfall and storm tides in this study, which was adopted as input boundaries of the urban hydrodynamic model.

When establishing the copula model, the first step is to determine marginal distributions of rainfall and storm tides. Commonly used methods for acquiring marginal distributions include parametric and non-parametric methods [18]. The parametric method has been used in much research [14,19,20], and it requires the assumption that the data (i.e., rainfall, storm tide) come from a known family of parametric distributions (e.g., Generalized Extreme Value distribution, Gumbel distributions, etc.). The non-parametric approach (e.g., kernel density estimation) has the advantage of not assuming a specific distribution and can directly use the data information to obtain the marginal distribution [21]. Different parameter estimation methods have different abilities to describe rainfall and storm tide distributions, especially for extreme values [22]. However, extreme rainfall and storm tides are of particular concern in the management of urban flood risk. To our knowledge, few studies have focused on the influence of parameter estimation methods on the flood risk of rainfall and storm tides.

In addition, urban flooding facilities are usually designed to resist the hazards with specific return period (e.g., 20 years or 50 years, etc.). For example, municipal pipe networks are designed to withstand 20-year rainstorms for particularly important inland cities in China. For the coastal city, rainfall and storm tides are both the hazard factors for urban floods. The bivariate return periods (RPs) of rainfall and storm tides are commonly described by the co-occurrence RP (i.e., “AND” scenario) [11,23], joint RP (i.e., “OR” scenario) [24,25] and Kendall RP [15,26]. However, which one is more suitable as the design standard for urban flooding facilities? What is the difference in flood risks caused by the rainfall and storm tides under different types of return periods? Tackling these problems is meaningful for urban flooding management and flooding facility design in coastal cities.

Therefore, the objectives of the study are to: (1) investigate the flood risk affected by the interaction of rainfall and storm tides coupling statistical and hydrodynamic models in the coastal city; (2) evaluate the influence of parametric and non-parametric methods of statistical models on the bivariate design and flood risk of rainfall and storm tides; (3) quantify the difference in the flood risk obtained by different types of bivariate RPs, and recommend the suitable one for different application needs. It is envisaged that the results would provide sufficient information to more accurate estimate the flood risk in the coastal areas.

## 2. Methods

**Figure 1:**
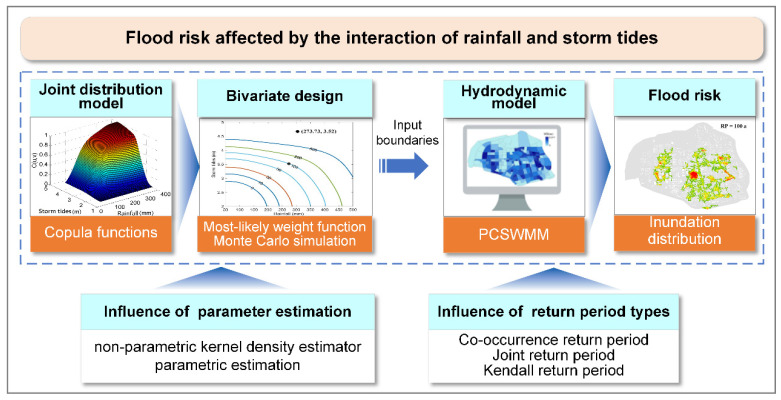
The research framework of the study.

In the study, flood risks influenced by the compound effect of rainfall and storm tides are investigated based on statistical and hydrodynamic models. The statistical model is used to obtain the bivariate design of rainfall and storm tides with the integration of copula function, most-likely weight function and Monte Carlo simulation method. The hydrodynamic model established by Personal Computer Stormwater Management Model (PCSWMM) is adopted for flood risk assessment, and the input boundary of the model is determined from the bivariate design of rainfall and storm tides. Finally, the influence of different parameter estimation methods and bivariate return periods on the bivariate designs and flood risks are investigated. The research framework is shown in Figure 1.

### 2.1. Copula Model of Rainfall and Storm Tides

The copula function *C*(*u*_1_, *u*_2_, *…*, *u_n_*) is a connection function proposed by Sklar [27] that is being increasingly employed in multivariate analysis (e.g., flood and drought frequency analysis, etc.), which connects the joint distribution function {$F(x_{1},x_{2},\cdots ,x_{n})$} of random variables ($X_{1},X_{2},\cdots ,X_{n}$) with their respective marginal distribution functions ($F_{1},F_{2},\cdots ,F_{n}$):

(1) $$F(x_{1},x_{2},\cdots ,x_{n})=C(F_{1}(x_{1}),F_{2}(x_{2}),\cdots ,F_{n}(x_{n}))$$

The Archimedean copula family is more desirable for hydrologic analyses, because it can be easily constructed, a large variety of copula families belong to this family, and it can be applied whether the correlation amongst hydrologic variables is positive or negative [28,29]. In the study, three Archimedean copula functions (Table 1) were adopted to establish the bivariate joint distribution. The Kolmogorov–Smirnov (K–S) test method was used to test the goodness of fit. The Akaike information criterion (*AIC*) and ordinary least squares criteria (*OLS*) were used to select the optimal copula function.

### 2.2. Parameter Estimation Methods 

Parametric and non-parametric estimation methods were used to determine the marginal distribution. For parametric estimation, four commonly used univariate distribution functions (i.e., Lognorm, Gamma, Weibull and Generalized Extreme Value distributions) were adopted to fit the rainfall and storm tide distributions. The parameters were estimated by the maximum likelihood function. The above functions are described in Table 2.

For non-parametric estimation, the kernel density estimator adopted by Balbhadra et al. [30] was used to obtain the marginal distribution, which was described as follows.

For the sample $X_{1},X_{2},\cdots ,X_{n}$, the kernel density estimator at any point *x* is estimated as:

(2) $$\overset{\wedge }{f_{h}}(x)=\frac{1}{nh}\sum_{i=1}^{n}K\left(\frac{x-X_{i}}{h}\right)$$

where *K*() is the kernel function; and *h* is the bandwidth of the data.

At the same time, the kernel function *K*() is required to meet the following conditions:

(3) $$K\left(x\right)\geq 0,\int_{-\infty }^{+\infty }K\left(x\right)dx=1$$

There are many expressions of kernel function, such as Gaussian kernel function, Triangle kernel function, Uniform (or Box) kernel function, etc. The Gaussian kernel function is used in the study since it is the most widely used kernel function and has been extensively studied in hydrology fields [31,32,33]. The equation of the Gaussian kernel function is as follows:

(4) $$K\left(x\right)=\frac{1}{\sqrt{2\pi }}\exp\left(-\frac{x^{2}}{2}\right)$$

The bandwidth *h* is very important for kernel density estimation because the kernel estimator is highly sensitive to bandwidth [34]. Considering a large sample, the following formula is used to estimate the optimal bandwidth:

(5) $$\overset{\wedge }{h}={\left\{\frac{{\int \left[K\left(x\right)\right]}^{2}dx}{\sigma_{k}^{4}{\int \left[f^{\prime \prime }(x)\right]}^{2}dx}\right\}}^{\frac{1}{5}}n^{-\frac{1}{5}}$$

In particular, when the overall distribution obeys *N*(0, σ^2^) and the kernel function is Gaussian kernel function, the optimal bandwidth is as follows:

(6) $$\overset{\wedge }{h}={\left(\frac{4}{3}\right)}^{\frac{1}{5}}\sigma n^{-\frac{1}{5}}\approx 1.06\sigma n^{-\frac{1}{5}}$$

### 2.3. Bivariate Return Period 

#### 2.3.1. Joint Return Period

Suppose the joint distribution function of rainfall (*H*) and storm tides (*Z*) is F(h,z), and the marginal distribution function is $F_{H}(h)$ and $F_{Z}(z)$. The probability that one of the variables of *H* and *Z* exceeds a certain magnitude is the joint probability P∪(h,z), and the corresponding return period is the joint return period T∪(h,z):

(7) $$T\cup (h,z)=\frac{1}{P\cup (h,z)}=\frac{1}{P((H>h)\cup (Z>z))}=\frac{1}{1-F(h,z)}$$

#### 2.3.2. Co-Occurrence Return Period

The probability that the *H* and *Z* both exceed a certain magnitude is the co-occurrence probability P∩(h,z), and the corresponding return period is the co-occurrence return period T∩(h,z):

(8) $$T\cap (h,z)=\frac{1}{P\cap (h,z)}=\frac{1}{P\left(\left(H>h)\cap (Z>z\right)\right)}=\frac{1}{1-F_{H}(h)-F_{Z}(z)+F(h,z)}$$

#### 2.3.3. Kendall Return Period

**Figure 2:**
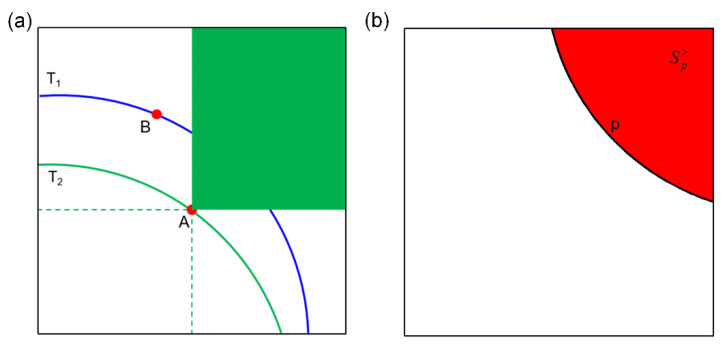
Graphical illustration of the dangerous region in (**a**) co-occurrence RP case; and (**b**) Kendall RP case.

The traditional multivariate return period (i.e., joint RP, co-occurrence RP) may have a deviation in the identification of the dangerous region [15,26,35]. For instance, Figure 2a presents the graphical illustration of the dangerous region in the co-occurrence RP case. The blue and green lines represent the isoline of RP T_1_ and T_2_, respectively. For a given realization A lying on the isoline of level T_2_, the green area means dangerous regions of event A. However, given another realization B, lying on the isoline of level T_1_ > T_2_, event B is more dangerous than event A obviously, but B does not belong to the dangerous regions of event A. To solve the problem, the Kendall RP was proposed by Salvadori et al. [26] based on Kendall distribution function (see Equation (10)). For Kendall RP, the event spaces are partitioned into the safety region $S_{p}^{<}=\left\{(u,v)\in R^{2}:C(u,v)<p\right\}$, the critical layer C(u,v)=p, and the dangerous region $S_{p}^{>}=\left\{(u,v)\in R^{2}:C(u,v)>p\right\}$ (red area in Figure 2b). Therefore, the dangerous region with a small *p* value will definitely cover the dangerous region with a larger *p* value, thus avoiding wrong identification of the dangerous region. The Kendall RP can be defined as follows:

(9) $$T_{k}=\frac{1}{P[C\left(u,v\right)>p]}=\frac{1}{1-K_{c}\left(p\right)}$$

(10) $$K_{c}\left(p\right)=P[C\left(u,v\right)\leq p]=\frac{1}{n}\sum_{i=1}^{n}I\left(C_{i}\leq p\right)$$

where $K_{c}\left(p\right)$ is the Kendall distribution function [36]. The $p\in \left(0,1\right)$ is a probability level; *u* and *v* are marginal distributions of rainfall and storm tides; *C*(*u*,*v*) is the copula function; and *I*(·) is an indicator function that equals 1 when the expression is correct and equals 0 otherwise.

For Archimedean copulas, the analytical formula of $K_{c}$ can be solved by the following formula:

(11) $$K_{c}(p)=p-\frac{\varphi (p)}{\varphi^{\prime }(p^{+})}$$

where φ(p) is the generator of the copula function; and $\varphi^{\prime }(p^{+})$ is the right derivative of φ(p). The generators φ of Archimedean copulas are shown in Table 1.

### 2.4. Most-Likely Weight Function Method

For univariate design, a return period corresponds to only one design value. However, for bivariate design, a given bivariate RP actually corresponds to many different combinations of rainfall and storm tides [37,38]. Therefore, it is difficult to select a suitable combined value for a specific bivariate RP. To solve the problem, Salvadori et al. [26] proposed the most-likely weight function to determine the bivariate design value based on the maximum product of the joint and marginal probability densities, indicating that the combination has the highest occurrence probability [38]. It has been widely used in multivariate design of rainstorm [39,40], flood [41,42] and drought [38,43] in recent years. Although the maximum possible weight function method may also have some disadvantages, such as the most likely event is not always the most potentially risky, the most-likely weight function method was adopted for the design of precipitation and storm tides since no uniform criteria are available to guide the selection of the appropriate combinations. The calculation formula of the most-likely weight function method is as follows:

(12) $$\left(h_{m},z_{m}\right)=\arg\max f(h,z)$$

(13) $$f(h,z)=c\left(u_{h},v_{z}\right)f_{H}\left(h\right)f_{Z}\left(z\right)$$

(14) $$h_{m}=F_{H}^{-1}(u_{h})$$

(15) $$z_{m}=F_{Z}^{-1}(v_{z})$$

where (*h*_m_, *z*_m_) is the selected combination for the bivariate design against flooding; $c\left(u_{h},v_{z}\right)$ is the probability density function of the copula function; and $f_{H}\left(h\right)$ and $f_{Z}\left(z\right)$ are the probability density functions of rainfall and storm tides, respectively. $F_{H}^{-1}(u_{h})$, $F_{Z}^{-1}(v_{z})$ are the inverse functions of the marginal distribution.

The steps of bivariate design by the most-likely weight function method are as follows:

(1) When the marginal and joint distributions of rainfall–storm tides are determined, the Monte Carlo simulation method is adopted to simulate *n*_1_ sets of rainfall–storm tide combinations, and *n*_1_ is greater than 10,000 to ensure that the number of samples is large enough;
(2) Use Equations (7)–(9) to calculate the return period of each combination. For a given return period *T*, select all combinations $\left(u_{h},v_{z}\right)$ with the return period of *T*;
(3) Calculate a combination $\left(u_{h},v_{z}\right)$ that makes f(h,z) reach the maximum by Equation (13);
(4) Finally, calculate the combined design of rainfall and storm tides (*h*_m_, *z*_m_) based on the inverse function of the marginal distribution (Equations (14) and (15)).

### 2.5. Urban Hydrodynamic Model

In the study, PCSWMM was used to simulate the flooding of the coastal city, which was developed by Computational Hydraulics International (CHI), Canada. PCSWMM made up for the defect that SWMM could only be used to simulate one-dimensional pipeline and river flow, but not two-dimensional surface flooding distribution, and it was widely adopted in urban flooding simulation [44,45,46,47,48]. The continuity and momentum equations used in PCSWMM are expressed as follows:

(16) $$\frac{\partial A}{\partial t}+\frac{\partial Q}{\partial l}=0$$

(17) $$\frac{\partial Q}{\partial t}+\frac{\partial (Q^{2}/A)}{\partial l}+gA\frac{\partial H}{\partial l}+gAS_{f}+gAh_{L}=0$$

where *A* is the cross-sectional area, m^2^; *Q* is the flow, m^3^/s; *t* is the time, s; *l* is the distance along the conduit, m; *S_f_* is the friction slope; *g* is the gravity acceleration, m/s^2^; *H* is the pressure head, m; and *h_L_* is the local energy loss, m. In this study, the infiltration process was simulated by the Horton model, and the hydraulic process of rivers and conduits was calculated by the dynamic wave method [49]. The 1D conduit model and 2D floodplain model are integrated by the orifice connection method. The urban hydrodynamic model of the study area was introduced and calibrated in our previous studies [44].

## 3. Study Area and Data

**Figure 3:**
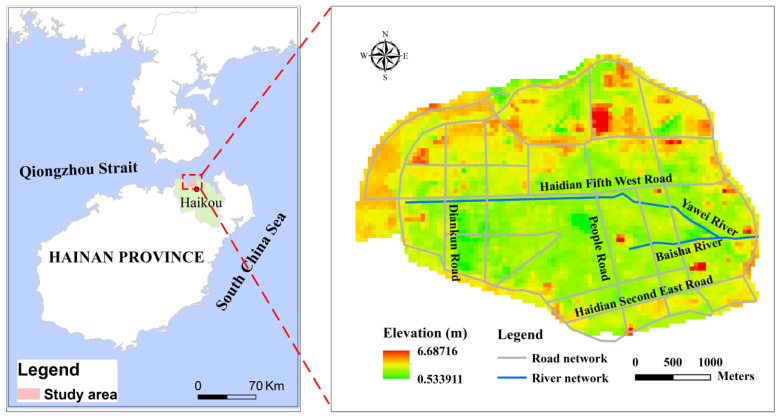
Study area.

The study area is located in the Haidian Island of Haikou, China (Figure 3). Haikou is the capital city of Hainan Province, which is a key free trade port built by the Chinese government in the northwestern part of the South China Sea. It is one of regions most frequently and seriously affected by tropical cyclones in China. Heavy rainfall and high storm tides are likely to occur simultaneously due to tropical cyclones and lead to severe floods in the study area. 

The study data include daily rainfall and storm tides with the length of 39 years provided by the Haikou Municipal Water Authority, which are used to establish copula models. The data for hydrodynamic models include DEM, conduit, inspection well, river section, construction distribution and historical inundation data. The DEM was obtained from http://www.resdc.cn/Default.aspx (accessed on 1 May 2022). The conduit and inspection well data were provided by Haikou Municipal Water Authority. The historical inundation data were accessed from through field investigation, and the construction distribution was extracted from the satellite remote sensing image. 

## 4. Results and Discussion

### 4.1. Bivariate Joint Distribution Model of Rainfall and Storm Tides

#### 4.1.1. Marginal Distribution Model

The annual maximum daily rainfall and its corresponding daily maximum storm tides were selected for the marginal distribution model. The parametric estimation and non-parametric kernel density estimation were used to determine the marginal distribution of rainfall and storm tides. 

**Figure 4:**
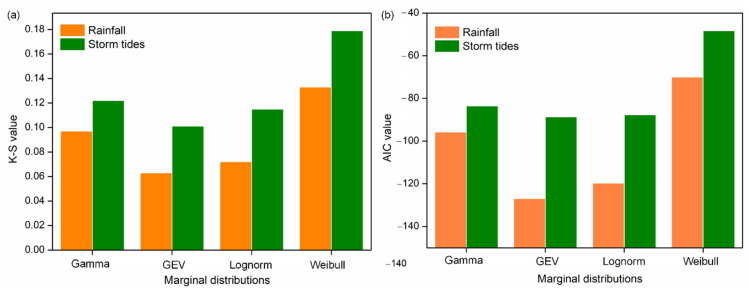
The (**a**) K–S and (**b**) *AIC* values of four marginal distributions.

**Figure 5:**
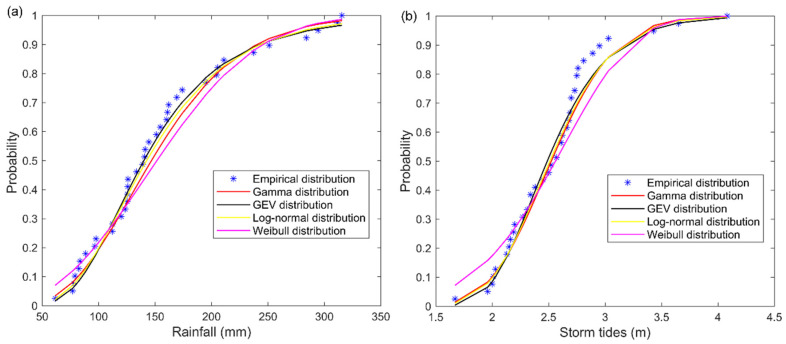
The empirical distribution and four marginal distributions of (**a**) rainfall, (**b**) storm tides.

For the parametric estimation, Lognorm, Gamma, Weibull and GEV distributions were selected to fit the marginal distributions. The above distributions all passed the K–S test with the significance level of 0.01. The K–S and *AIC* values of four marginal distributions are shown in Figure 4. The GEV distribution was chosen as the best model for rainfall and storm tides with the minimum *AIC* values. The fit of four marginal distributions are presented in Figure 5, and the parameters of GEV function are shown in Table 3. 

**Figure 6:**
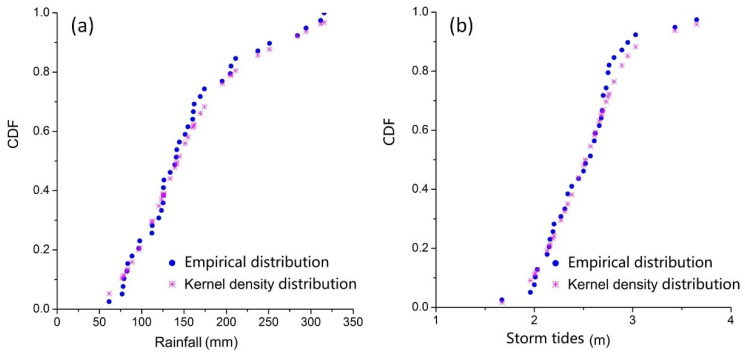
The empirical distribution and kernel density estimation distribution of (**a**) rainfall, (**b**) storm tides.

For the non-parametric method, the kernel density estimation was adopted for determining the marginal distributions of rainfall and storm tides. The K–S test statistic values of rainfall and storm tides are 0.069 and 0.097, respectively, which are less than the critical statistic value of 0.26 with the significance level of 0.01, indicating that the non-parametric kernel density estimation results pass the K–S test. Figure 6 shows the comparison between kernel density estimation distributions and empirical distributions, and the correlations are both more than 0.95, indicating that the kernel density estimation distributions are reasonable.

#### 4.1.2. Bivariate Joint Distribution Model

**Figure 7:**
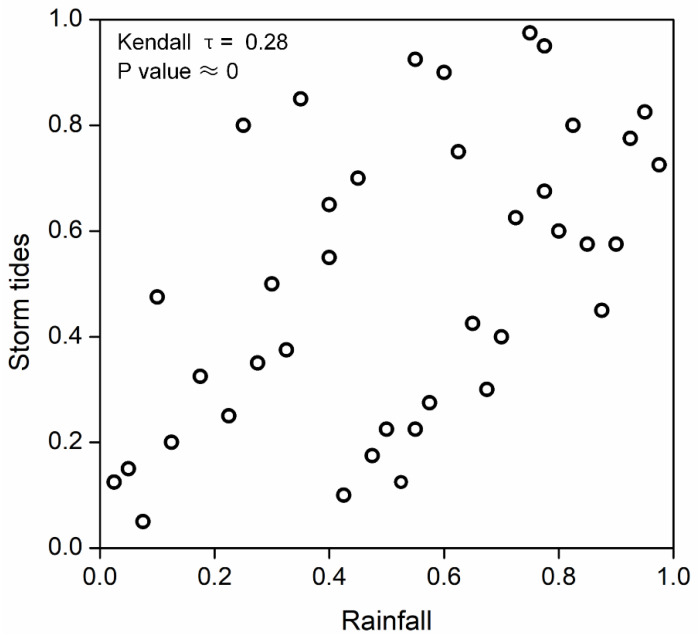
Bivariate rank-plots of the marginals of precipitation and storm tides.

The relationship between rainfall and storm tides was measured firstly before using the copula function to establish the joint distribution model. In the study, we adopted the normalized rank scatterplot used in Salvadori et al. [26] and Gräler et al. [50] to investigate the joint behavior of the variables. As presented in Figure 7, evidently, there are significant positive associations between rainfall and storm tides with the estimates of the Kendall’s τ = 0.28, and the corresponding *p*-values are negligible. Although the dependence is relatively low, it has an important impact on the flood risk of coastal areas [10,19,51]. Therefore, the dependence structure between rainfall and storm tides should be fully considered for compound flooding risk. In the study, a bivariate joint distribution model based on copula functions is conducted to describe the dependence structure of rainfall and storm tides.

For parametric estimation, three Archimedean copulas were used to establish the joint distribution model of rainfall and storm tides based on the maximum likelihood method, and the GEV function was adopted as the marginal distribution (mentioned in Section 4.1.1). All copulas pass the K–S test with the significance level of 0.01. The *AIC* values of the Clayton, Frank and Gumbel copulas are −92.778, −101.36 and −107.36, respectively, and the Gumbel copula has the smallest *AIC* value. Therefore, the Gumbel copula is well fitted to the joint distribution of rainfall and storm tides. 

**Figure 8:**
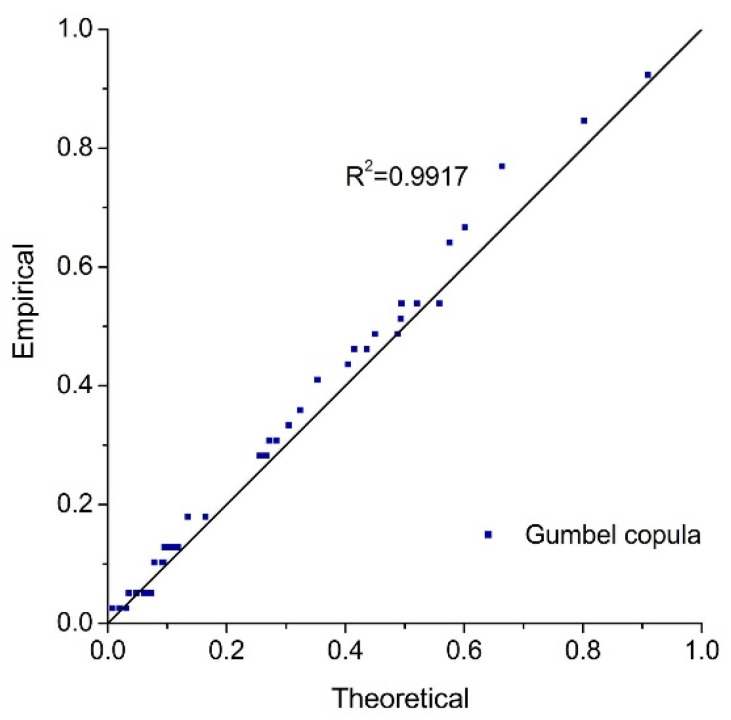
Probability–Probability (P–P) plot of Gumbel copula (non-parametric estimation method).

**Figure 9:**
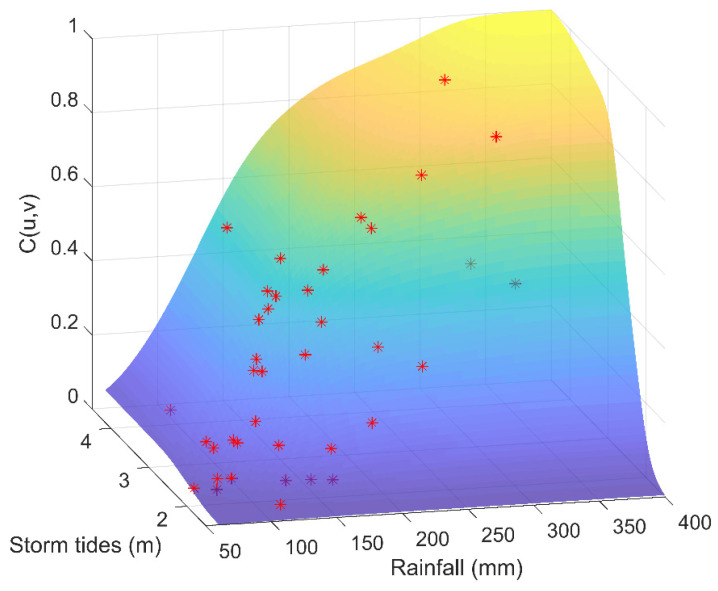
*C*(*u*,*v*) of rainfall and storm tides (non-parametric estimation method). Red dots are observed values.

For non-parametric estimation, marginal distributions obtained from the non-parametric kernel density estimation are adopted to conduct the joint distribution of rainfall and storm tides. The goodness-of-fit test is shown in Table 4. Three copulas all pass the K–S test with the significance level of 0.01. The Gumbel copula is the optimal model due to the minimum *AIC* and *OLS* values, which is consistent with the results of the parametric approach. Figure 8 and Figure 9 are the Probability–Probability (P–P) map and the joint probability distribution for the non-parametric approach, respectively, indicating that the selected copula is reasonable.

### 4.2. Influence of Parameter Estimation and RP Types on Bivariate Designs of Rainfall and Storm Tides

#### 4.2.1. The Influence of Parameter Estimation Methods 

**Figure 10:**
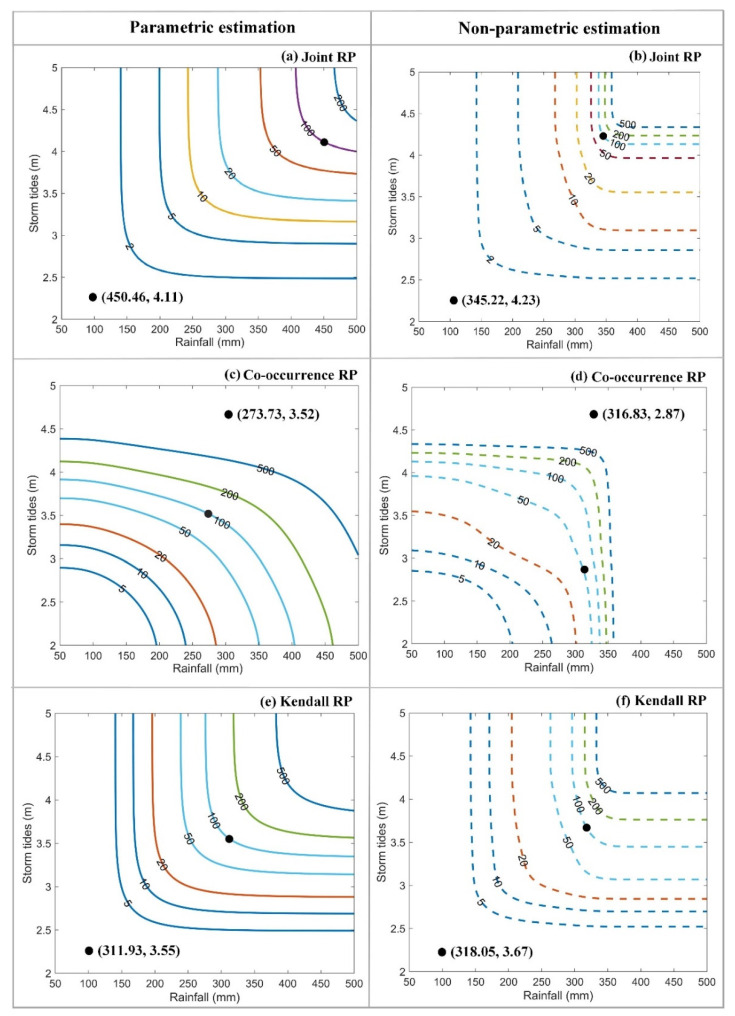
RP contour plots of rainfall and storm tides. (**a**,**c**,**e**) are the contour plots of joint RP, co-occurrence RP and Kendall RP obtained by the parametric estimation approach, respectively. (**b**,**d**,**f**) are the contour plots of joint RP, co-occurrence RP and Kendall RP obtained by the non-parametric estimation approach. Black points represent the bivariate design values of RP = 100 a.

The influence of parametric and non-parametric approaches on the bivariate design of rainfall and storm tides was investigated. The bivariate design was determined by integrating Gumbel copula and the most-likely weight function method. Figure 10 shows the bivariate RP contour plots and the design values of RP = 100 a (see black points). As demonstrated in Figure 10, we find that there are obvious changes in RP contour plots between parametric and non-parametric approaches, especially when the return period is large. For the parametric approach, the RP contour plots are evenly distributed with the RP increasing from 5 a to 500 a (Figure 10a,c,e). However, the contours are relatively denser when the RP is large (e.g., more than 100 a) for the nonparametric approach (Figure 10b,d,f), and the changes of design values are smaller than those of the parametric approach with increasing RP. For example, when the joint RP increases from 100 a to 200 a, the bivariate design value of rainfall and storm tides increases from (450.46 mm, 4.11 m) to (511.60 mm, 4.31 m) for parametric estimation, which are (345.22 mm, 4.23 m) and (354.49 mm, 4.31 m) for nonparametric estimation. The design value of rainfall only increases 9.27 mm for nonparametric estimation when the joint RP increases from 100 a to 200 a.

Therefore, although the non-parametric approach is a better fit for the observed data due to the smaller *AIC* values than that of the parametric approach, it may have the poor extrapolation capability, and when the design RP is large (e.g., more than 100 a) the design values may be underestimated, which is in line with the results of Huang et al. [35]. Besides, the length of the data has a significant influence on the non-parametric estimation, i.e., the kernel estimator may not provide accurate results if the sample is small. Therefore, the non-parametric estimation is recommended when the data length is sufficient (e.g., greater than 50 in Huang et al. [35] and Seaman et al. [52]). When the data length is small or the design RP is high, it is recommended to adopt the parametric approach for the bivariate joint distribution, so as to avoid low design values. 

#### 4.2.2. The Influence of RP Types 

As shown in Figure 10, the design value increases with the increase in RP, and the design value of the joint RP is the largest, with the middle of the Kendall RP and the smallest of co-occurrence RP. For example, the bivariate design value of rainfall and storm tides is (511.60 mm, 4.31 m) with the joint RP of 200 a, which is (356.83 mm, 3.75 m) and (318.93 mm, 3.71 m) for the Kendall RP and co-occurrence RP, respectively. 

The above difference is mainly caused by the difference in defining dangerous regions of joint, co-occurrence and Kendall RPs. As reported in Salvadori et al. [26], the dangerous regions may be enlarged or reduced for traditional bivariate RPs (i.e., co-occurrence RP, joint RP), resulting in unreasonable bivariate design values. Since the Kendall RP is determined based on the Kendall distribution function and points lying over the same critical layer C(u,v)=p generate the same dangerous region [15], it would avoid the limitation in the identification of safe and dangerous regions. Consequently, when there is no clear application requirement, the Kendall RP can be adopted for bivariate design standard of rainfall and storm tides. When flooding facilities need to deal with the simultaneous occurrence of rainfall and storm tides, the co-occurrence RP could be adopted as the bivariate design standard.

### 4.3. Compound Flood Risks with Different Designs of Rainfall and Storm Tides

**Figure 11:**
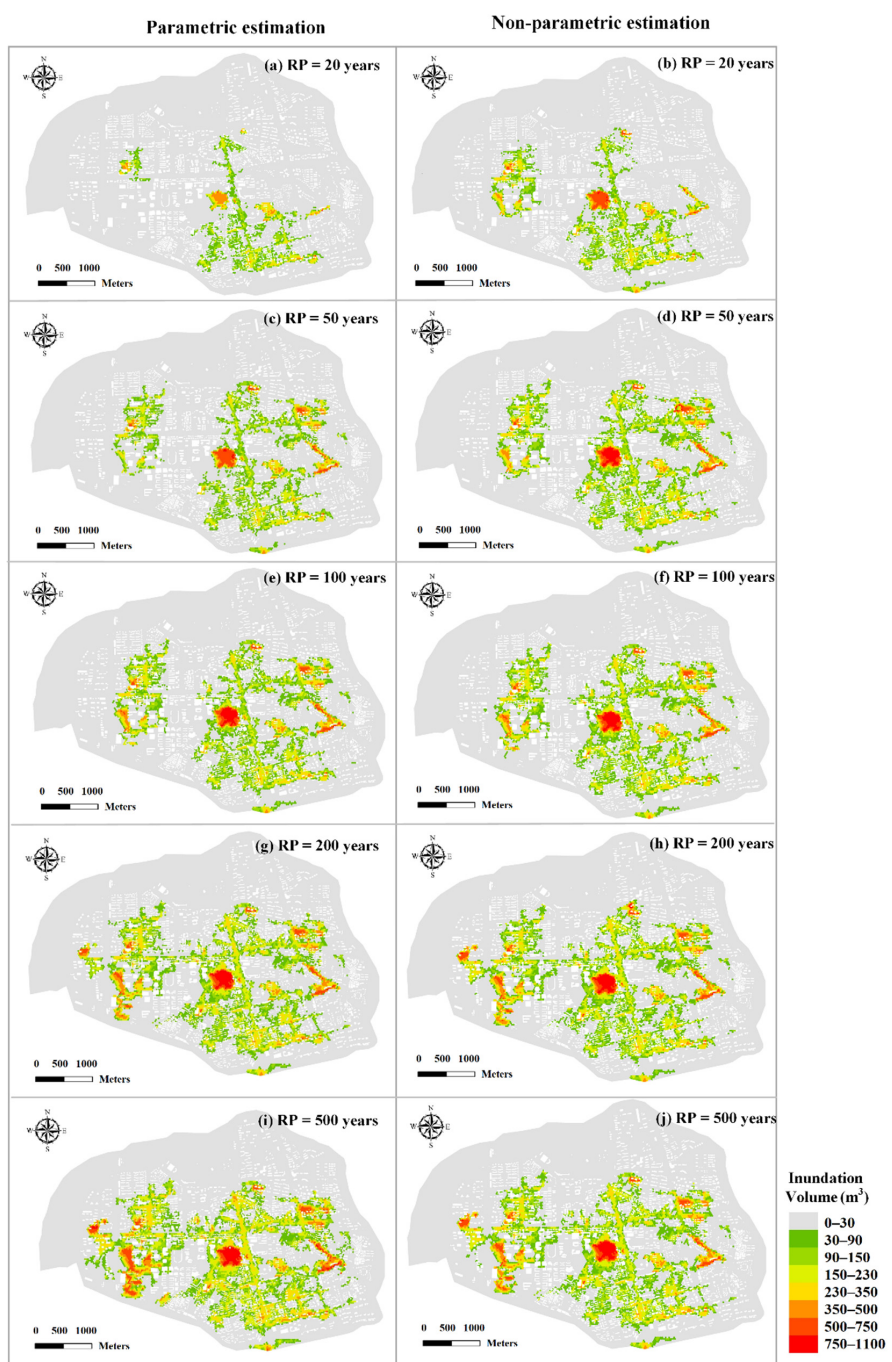
Flooding maps in different RPs for parametric and non-parametric estimation.

**Figure 12:**
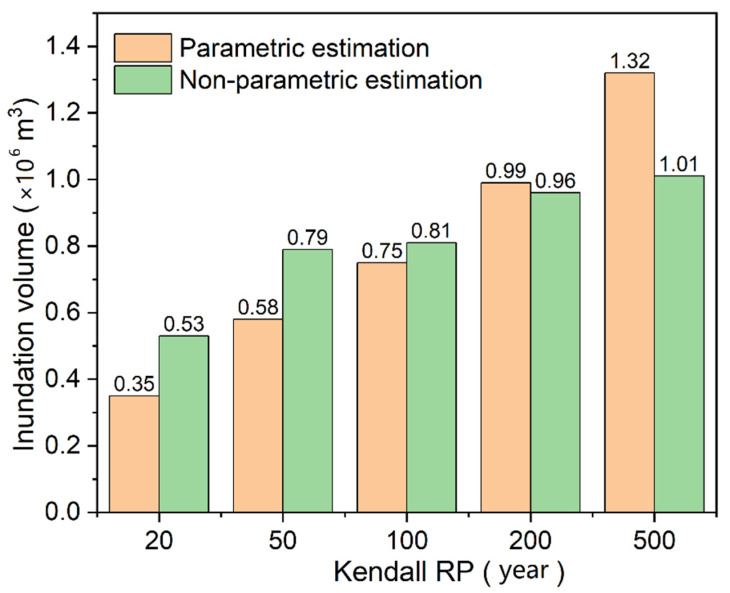
Comparison of inundation volumes for parametric and non-parametric estimation.

To evaluate flood risks of compound events with different RPs, the copula-based design values of rainfall and storm tides were adopted as the input boundaries of the urban hydrodynamic model. The flooding maps in different Kendall RPs are presented in Figure 11. The comparison of inundation volumes for parametric and non-parametric estimation are illustrated in Figure 12. As the Kendall RP increases from 20 a to 500 a, the inundation volume increases from 0.35 million m^3^ to 1.32 million m^3^ for parametric estimation of rainfall and storm tides, and 0.53 million m^3^ to 1.01 million m^3^ for non-parametric estimation. Overall, as demonstrated in Figure 11 and Figure 12, the flood risks in non-parametric estimation condition are more severe those that in parametric estimation condition when the Kendall RP is less than 100 a. However, when the Kendall RP is more than 100 a (e.g., 200 a and 500 a), the flood risk is much greater (with an average of 17%) in parametric estimation condition. Consequently, considering the most unfavorable scenario, parametric estimation approach is recommended when the design standard of flooding facilities is large.

**Figure 13:**
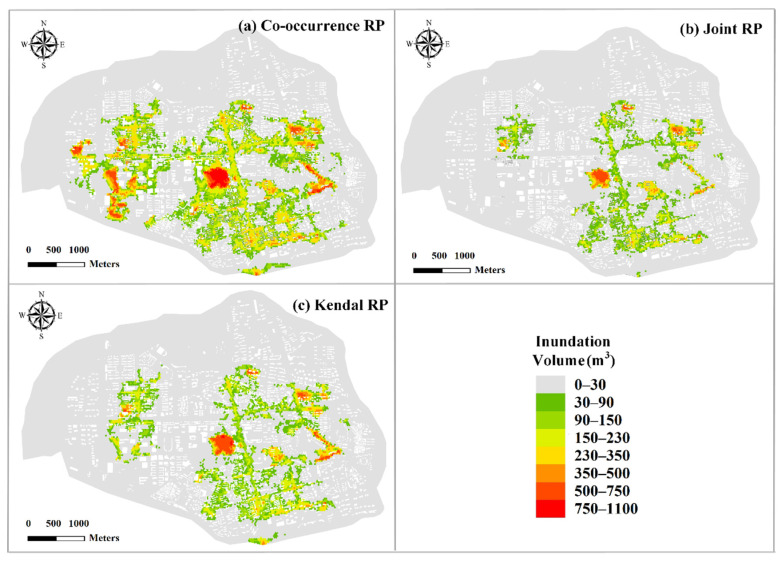
Comparison of inundation volumes for different types of bivariate RP.

In order to test the difference in flood risks under different types of bivariate RP, the flood risks of three bivariate RP types (i.e., co-occurrence RP, joint RP and Kendall RP) are evaluated with the RP of 50 a (see Figure 13). As shown in the figure, the flood risk varies considerably under different types of bivariate RP and is the highest for co-occurrence RP condition with the inundation volume of 11.7 million m^3^, which is 19 times higher than that for Kendall RP condition (0.58 million m^3^) and 29 times higher than that for joint RP (0.38 million m^3^). Therefore, the types of bivariate RP have the important impact on the flood risk, which is the highest under co-occurrence RP condition and the lowest under joint RP condition. Different types of bivariate RP can be used in different specific application needs. For instance, in case of interest in information about the flood risk caused by exceedance over either precipitation or storm tides, the joint RP scenario should be used, and the joint RP has been adopted in the studies of Bender et al. [53], Moftakhari et al. [25], and Ward et al. [9]. If the flood risk of simultaneous exceedances are considered, the co-occurrence RP should be adopted [54]. When there is no specific application need, we can employ the Kendall RP for bivariate design standard of rainfall and storm tides, which is consistent with Bender et al. [53].

## 5. Conclusions

Coastal cities are more vulnerable to floods due to the compound effect of rainfall and storm tides. In this study, the compound flooding risk is evaluated by the integration of the copula model, most-likely weight function and hydrodynamic model in a coastal city (Haikou, China). Moreover, the influence of two parameter estimation approaches (parametric and non-parametric kernel density estimator) for copula models and different bivariate RPs (co-occurrence, joint and Kendall RPs) on the flood risks are investigated. 

Compared to parametric approaches, the non-parametric approach is a better fit for the observed data, but the design values may be underestimated by an average of 17% due to its poor extension ability when the RP is more than 100 a. Therefore, the parametric estimation approach is recommended considering the most unfavorable scenario. The inundation risk is highly correlated with the selection of bivariate RP types. The Kendall RP can well describe the flood risk due to the reasonable definition of the dangerous areas for a multivariate scenario.

In this study, we established the joint distribution of rainfall and storm tides based on the copula function, but there is uncertainty in the parameters of both the marginal distribution and the copula function. One of the limitations of this paper is that the influence of parameter uncertainty on the copula function and flood risk is not considered, but will be our future research work. In addition, rainfall and storm tides show increasing trends under a changing environment. In the future, the non-stationary flood risk will be evaluated by the proposed framework in coastal cities.

## Figures and Tables

**Table 1:** Archimedean copulas and generators φ.

Copulas	*C*(*u*,*v*)	φ
Gumbel Copula	$C(u,v)=\exp\{-{\left[{\left(-\ln u\right)}^{\theta }+{\left(-\ln v\right)}^{\theta }\right]}^{1/\theta }\}$	$\varphi \left(t\right)={\left(-\ln t\right)}^{\theta }$
Clayton Copula	$C(u,v)={\left(u^{-\theta }+v^{-\theta }-1\right)}^{-1/\theta }$	$\varphi \left(t\right)=t^{-\theta }-1$
Frank Copula	$C(u,v)=-\frac{1}{\theta }\ln[1+\frac{\left(e^{-\theta u}-1)(e^{-\theta v}-1\right)}{e^{-\theta }-1}]$	$\varphi \left(t\right)=-\ln\left[\frac{e^{-\theta t}-1}{e^{-\theta }-1}\right]$

**Table 2:** The four commonly used univariate distribution functions.

Functions	*F*(*x*)	Parameters
Lognorm	$F(x)=\frac{1}{2}+\frac{1}{2}erf\left[\frac{\ln\left(x\right)-\mu }{\sqrt{2}\sigma }\right]$	*μ*, *σ*
Gamma	$F\left(x\right)=\int_{0}^{x}\frac{x^{\beta -1}}{\alpha^{\beta }\Gamma \left(\beta \right)}e^{-\frac{x}{\alpha }}dx$	*α*, *β*
Weibull	$F(x)=1-e^{-{\left(\frac{x-m}{a}\right)}^{b}},x-m>0$	*m*, *a*, *b*
Generalized Extreme Value (GEV)	$F(x)=\exp\left\{-{\left[1-k\left(\frac{x-\mu }{\alpha }\right)\right]}^{1/k}\right\},1-k\left(\frac{x-\mu }{\alpha }\right)>0$	*μ*, *α*, *k*

**Table 3:** Estimated values of marginal distribution parameters.

Distribution	Rainfall			Storm Tides		
	Shape Parameter *k*	Scale Parameter *σ*	Location Parameter *μ*	Shape Parameter *k*	Scale Parameter *σ*	Location Parameter *μ*
GEV	0.115	46.784	122.513	−0.048	0.380	2.349

**Table 4:** Test of the goodness of fit of *C*(*u*,*v*) (non-parametric estimation method).

Copula Function	K–S	*AIC*	*OLS*
Clayton Copula	0.123	−100.108	0.043
Frank Copula	0.113	−110.317	0.038
Gumbel Copula	0.106	−110.773	0.037

## Data Availability

Not applicable.
